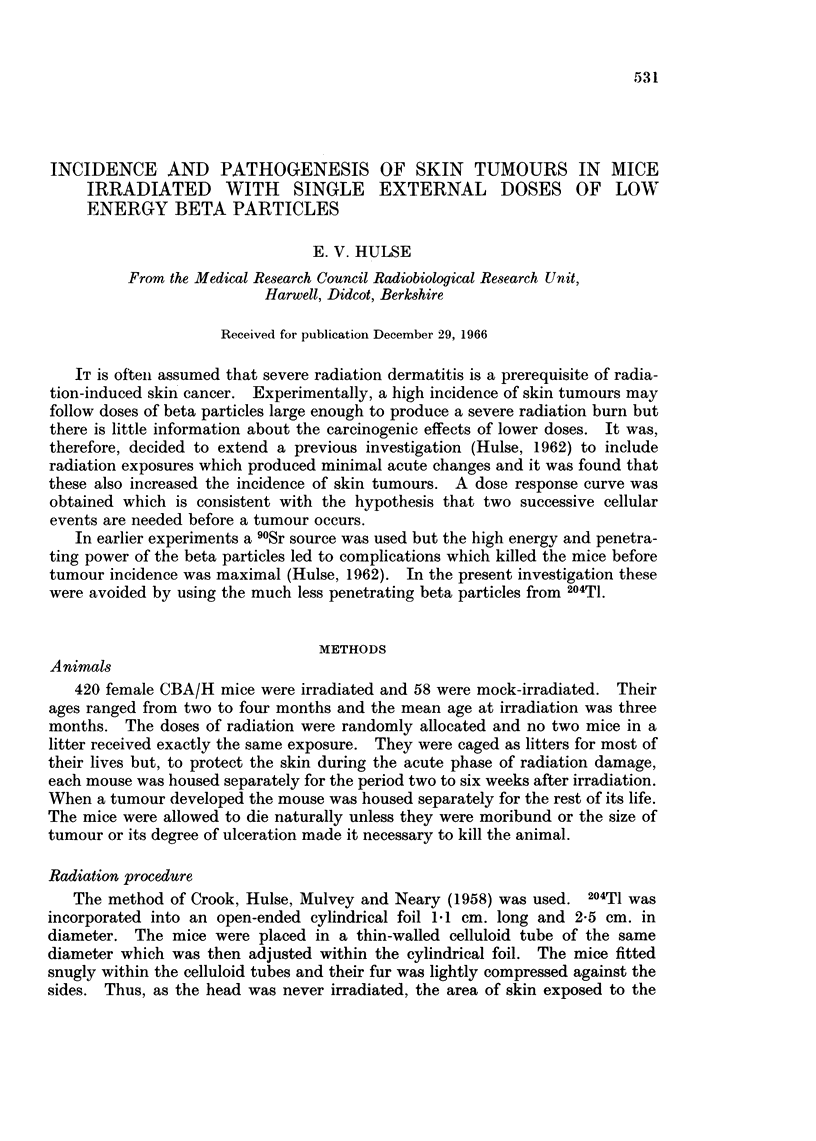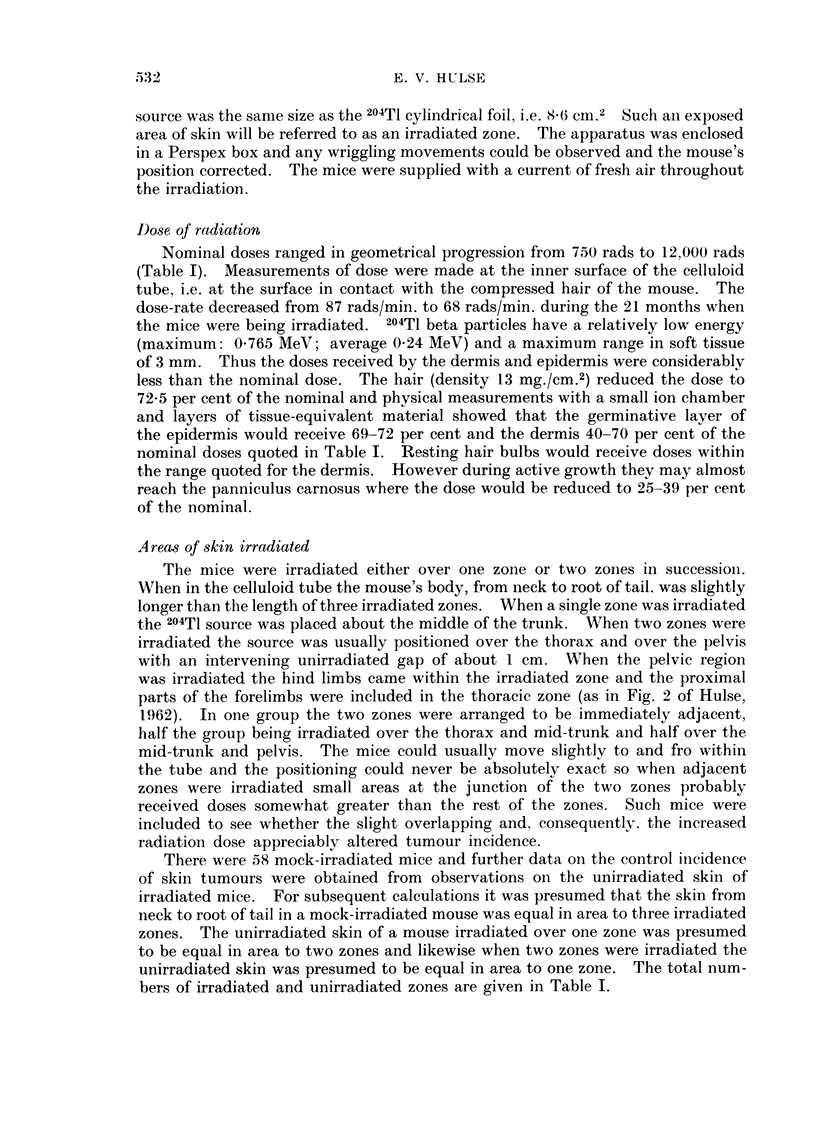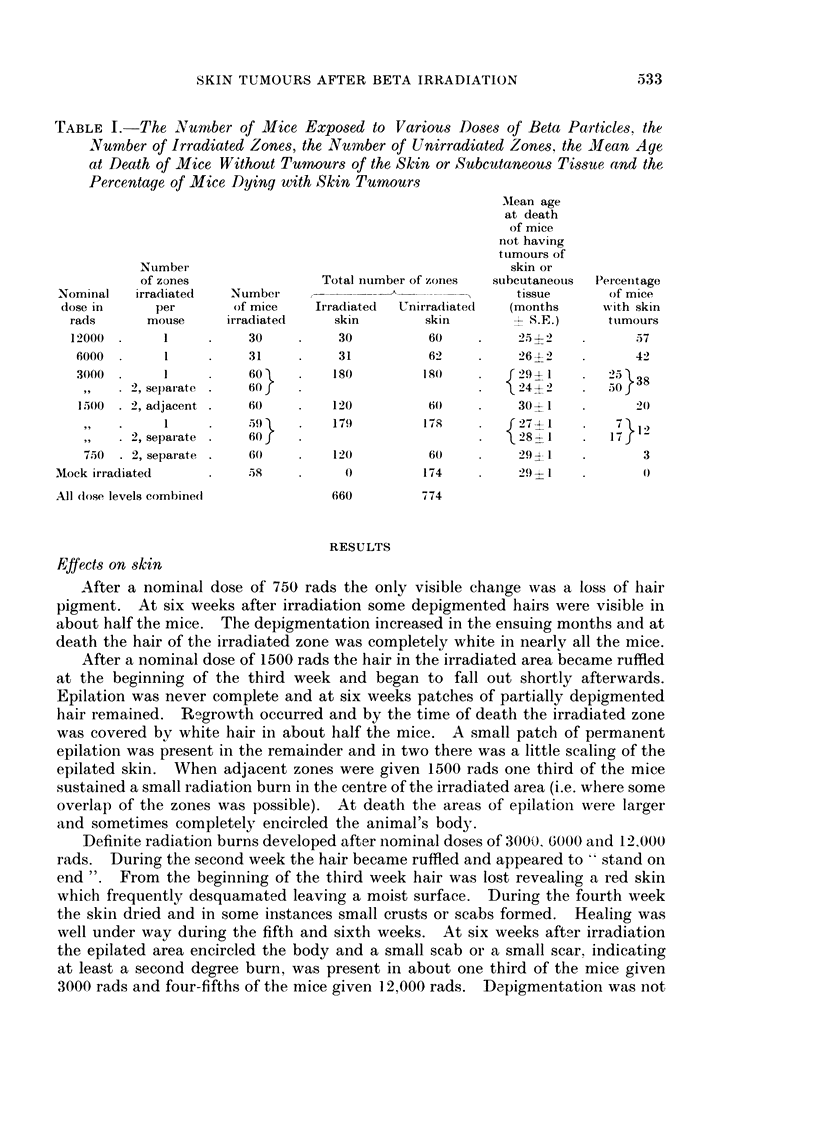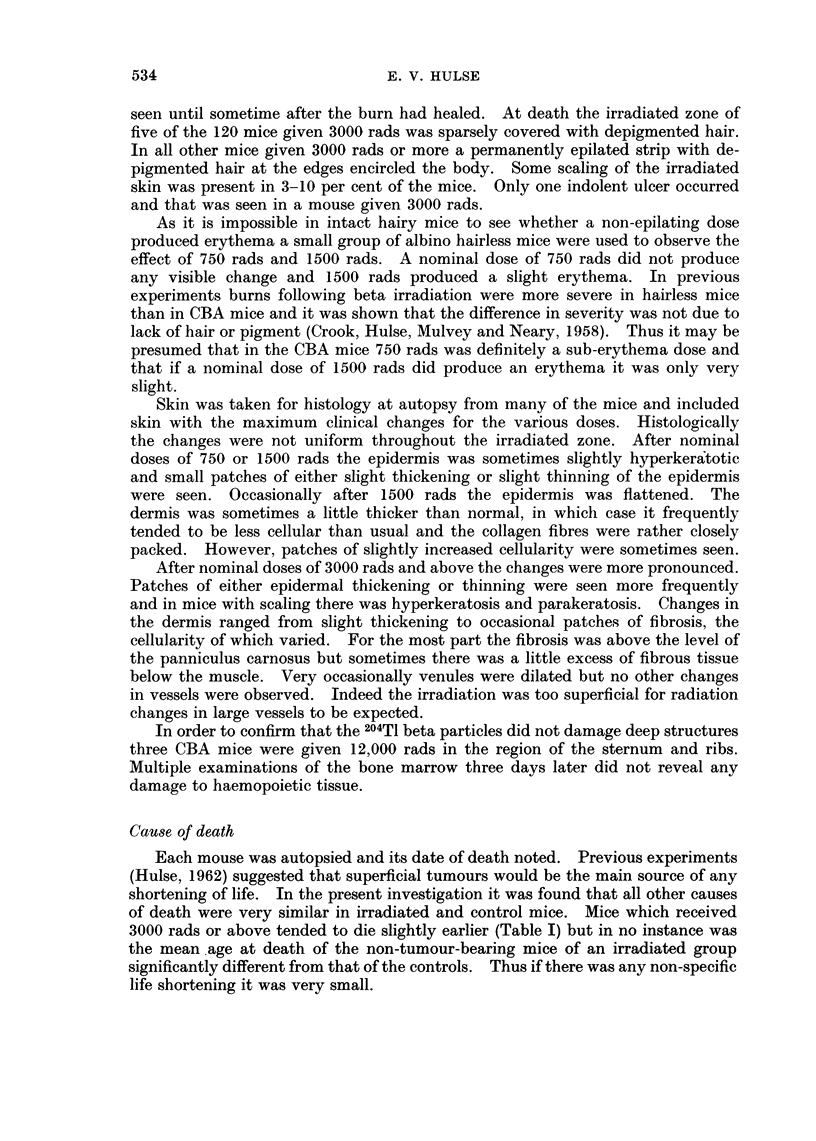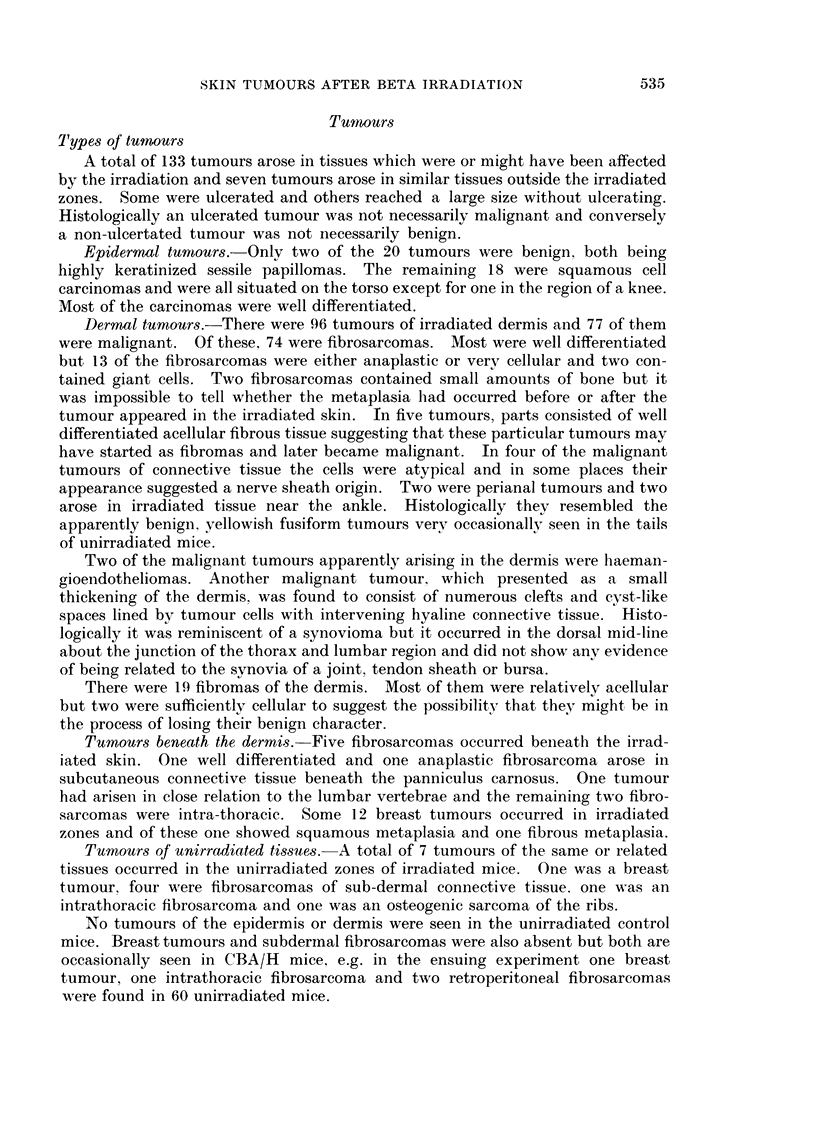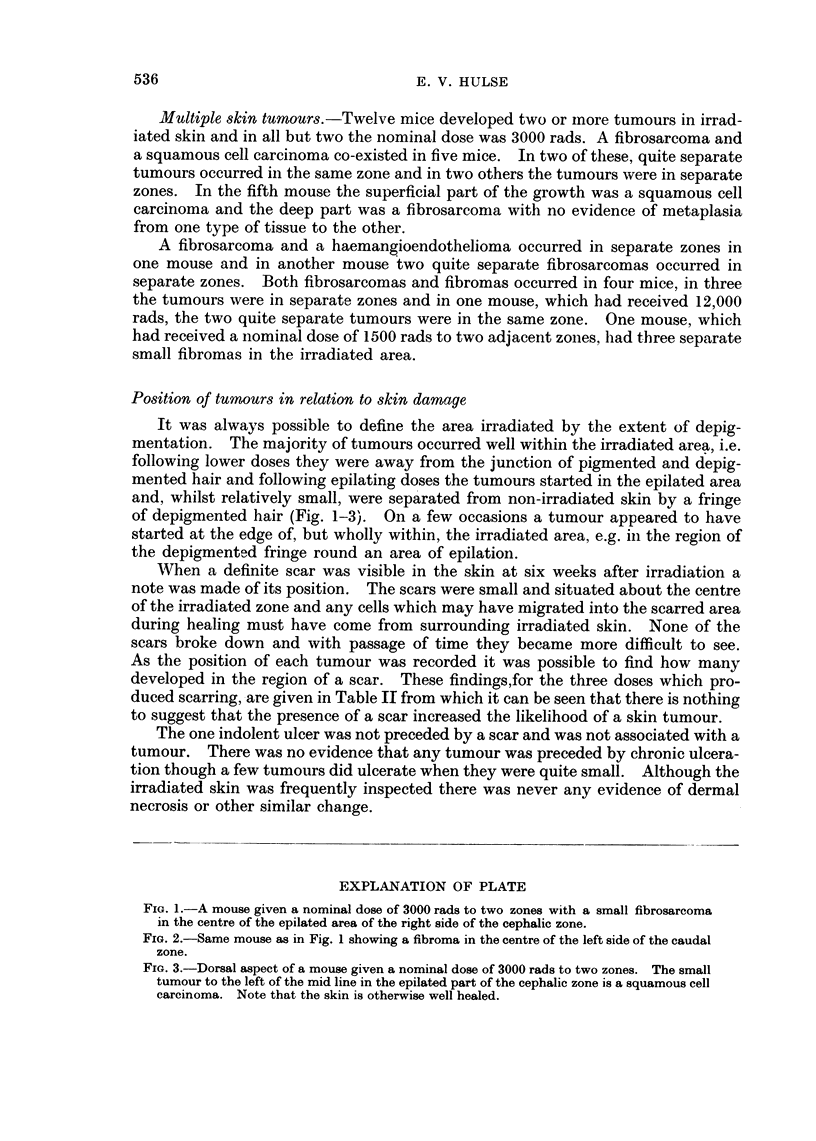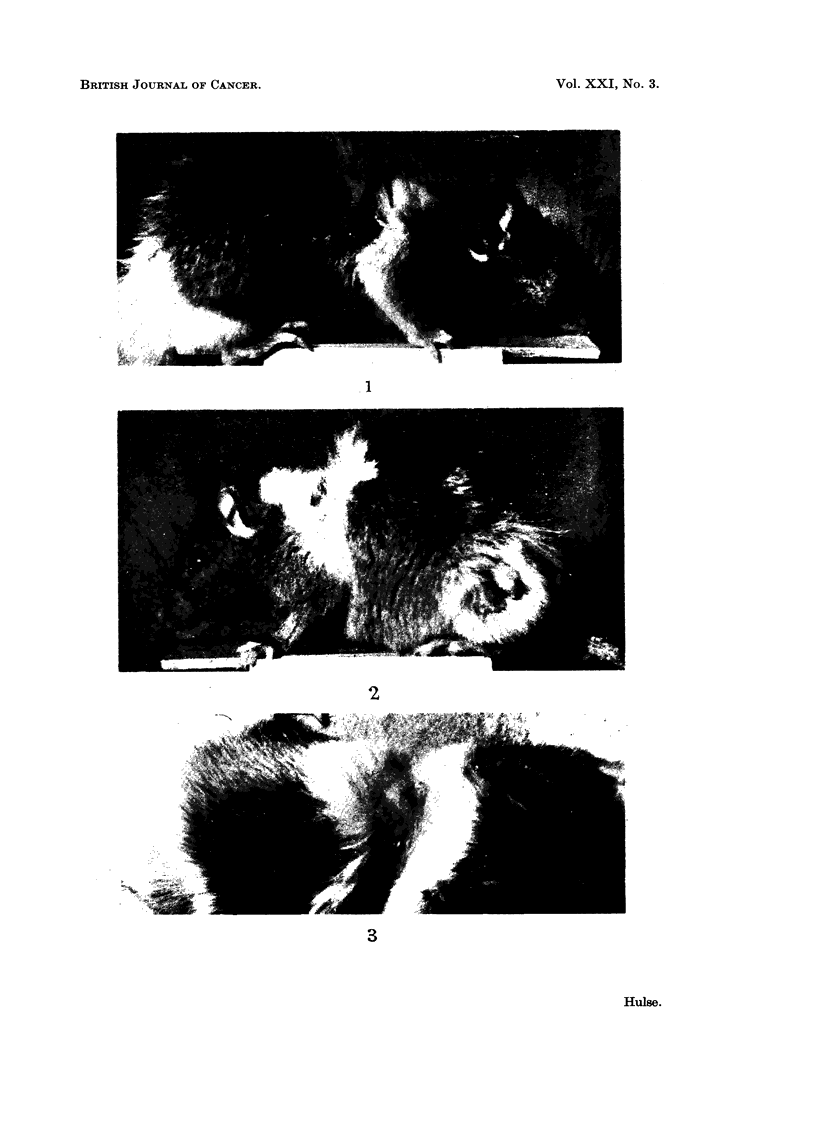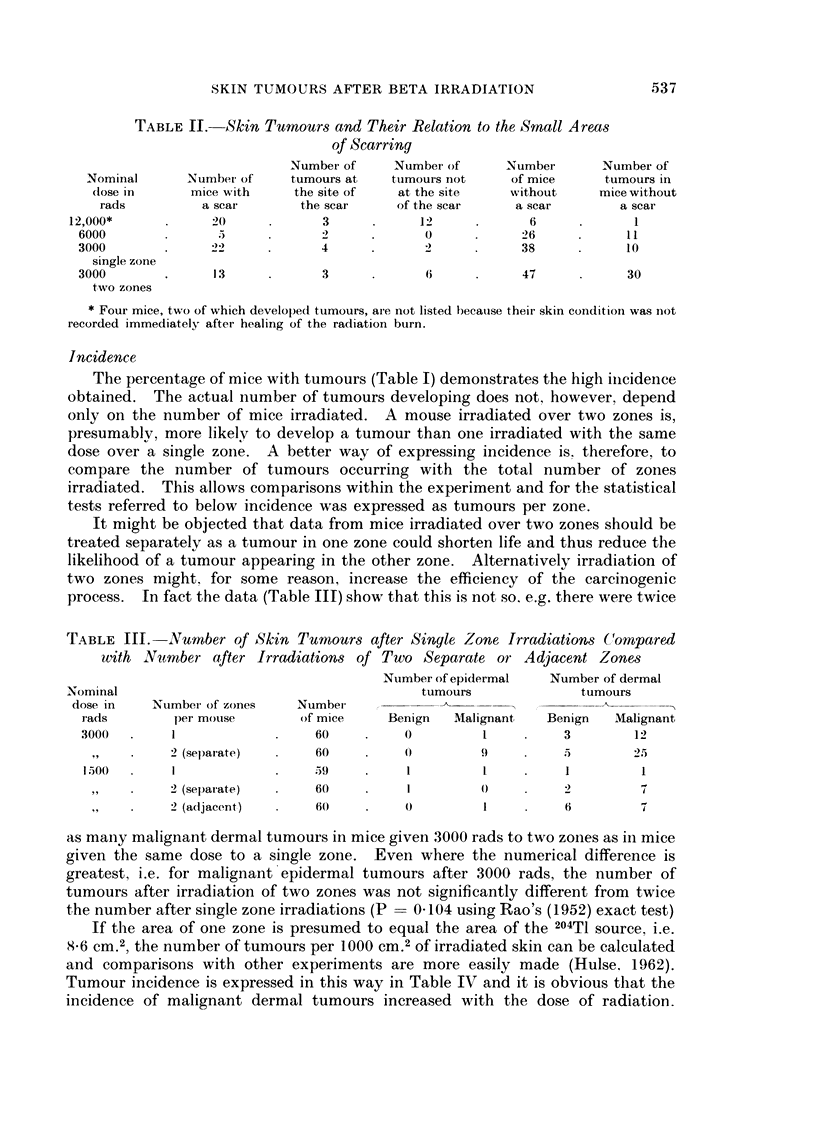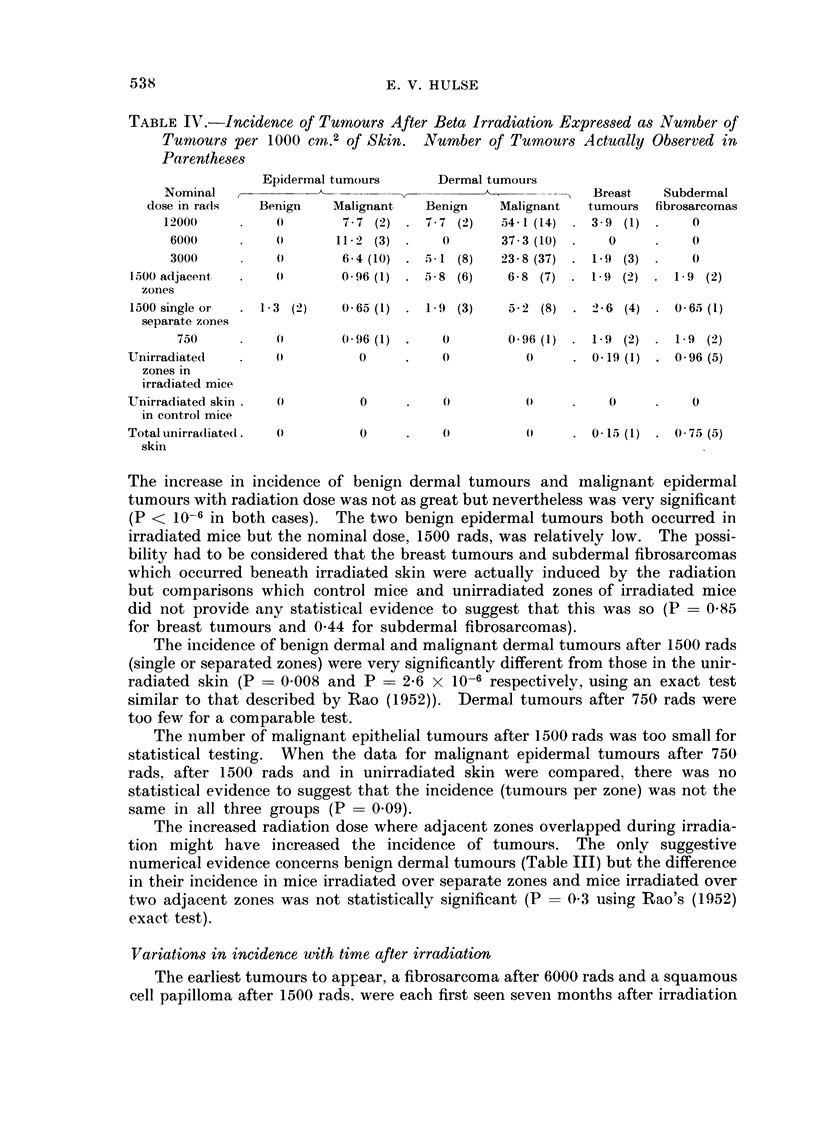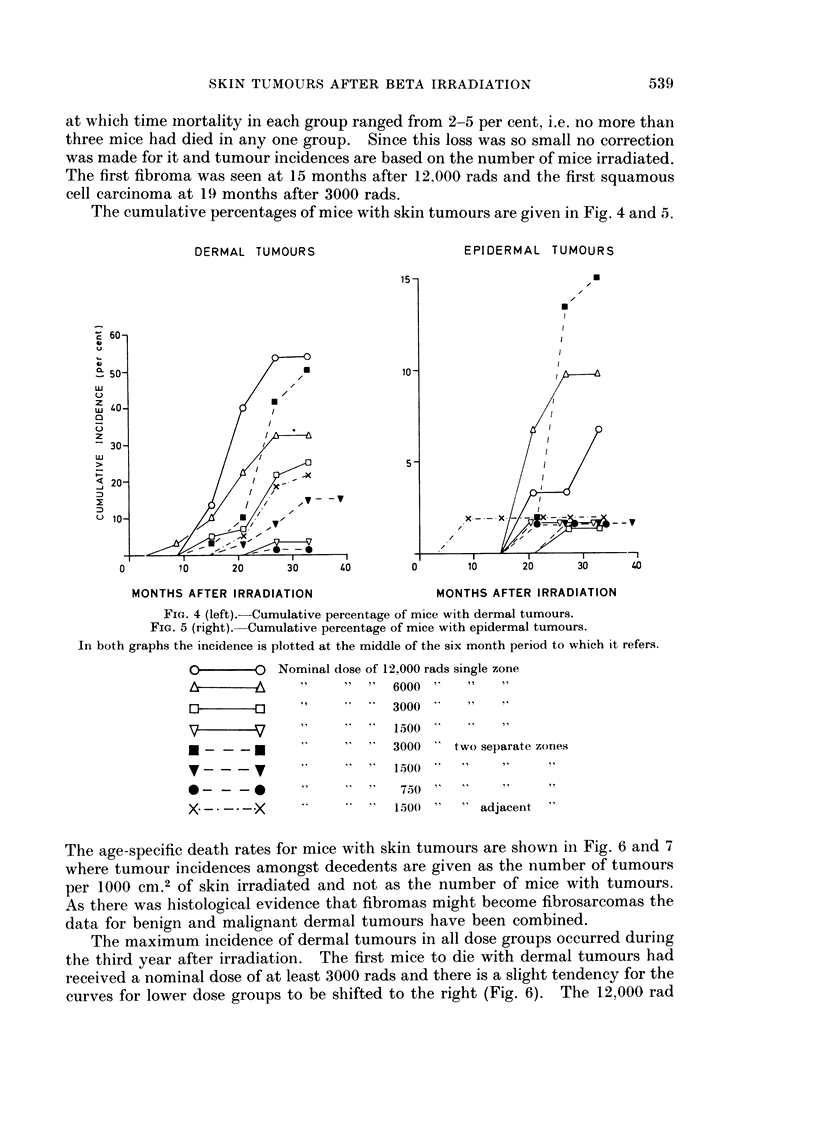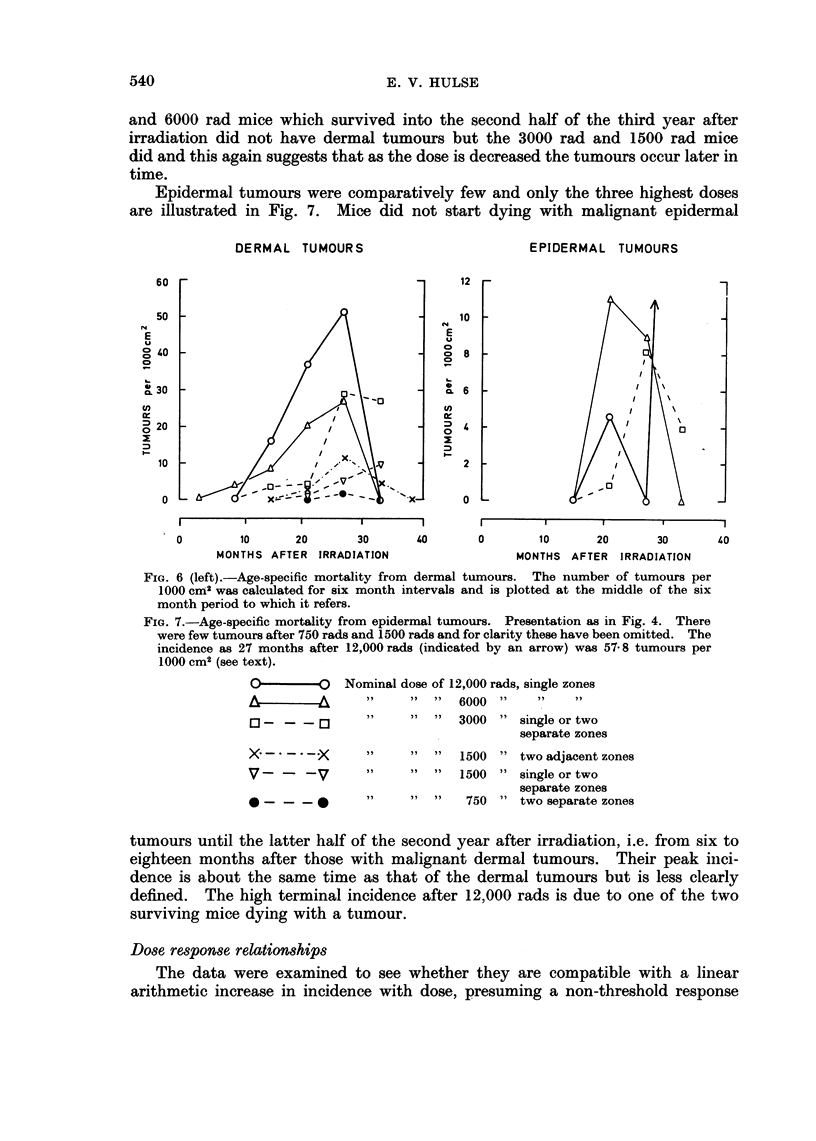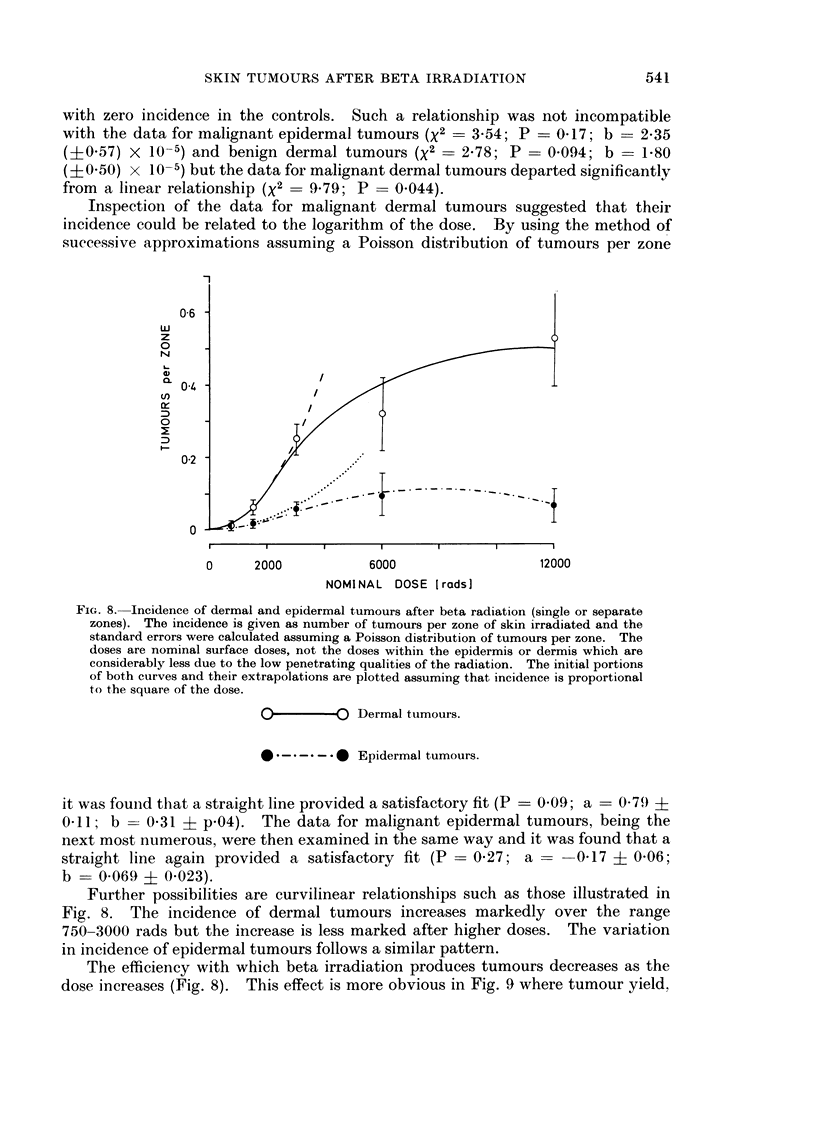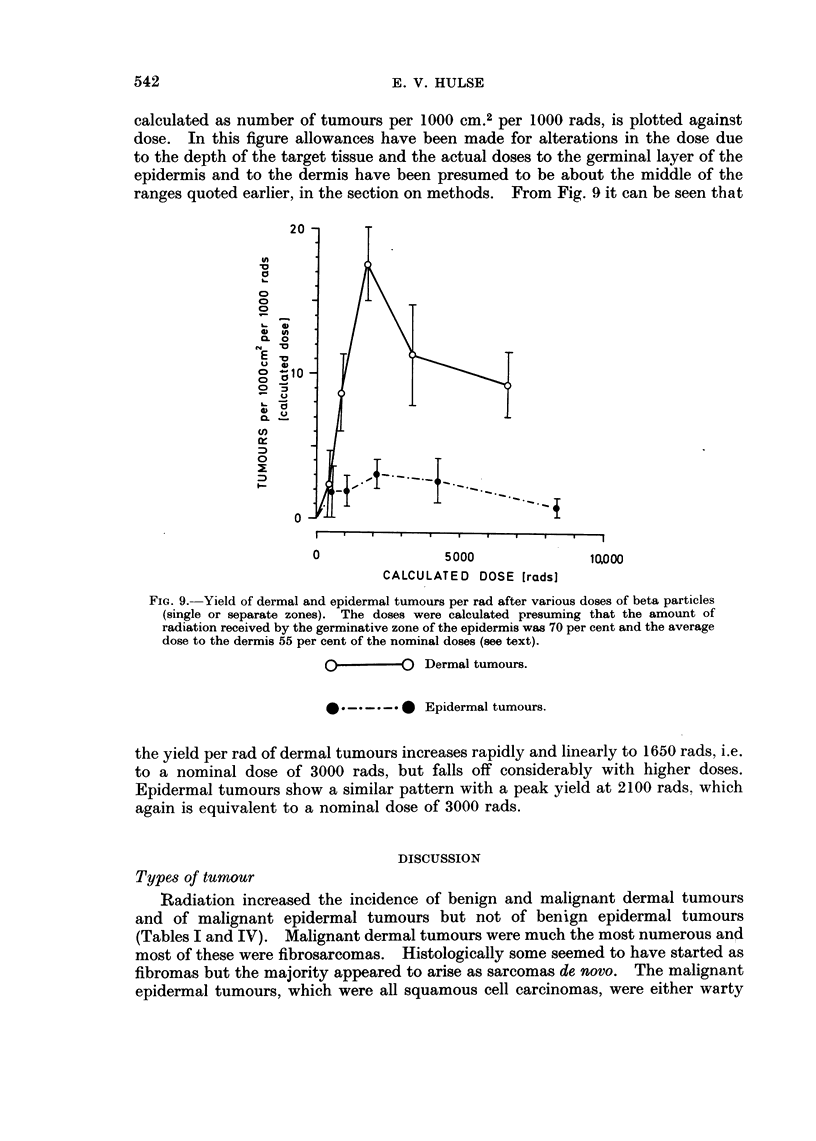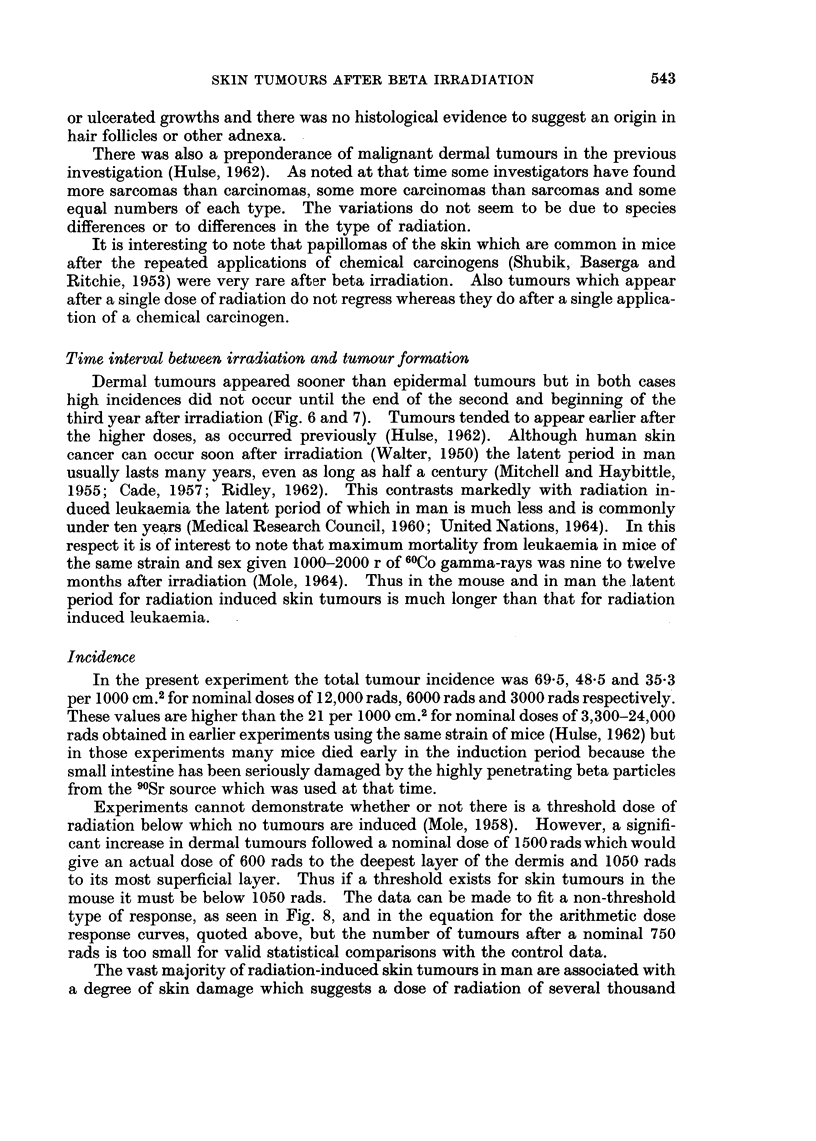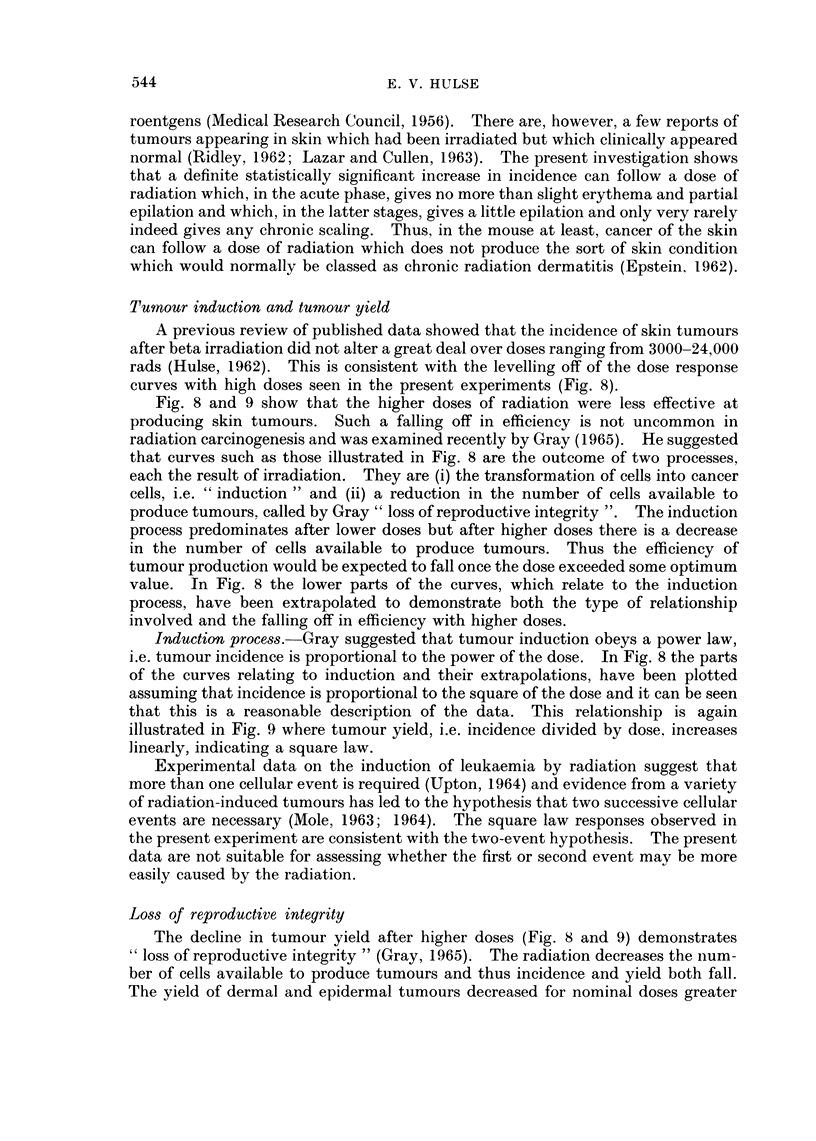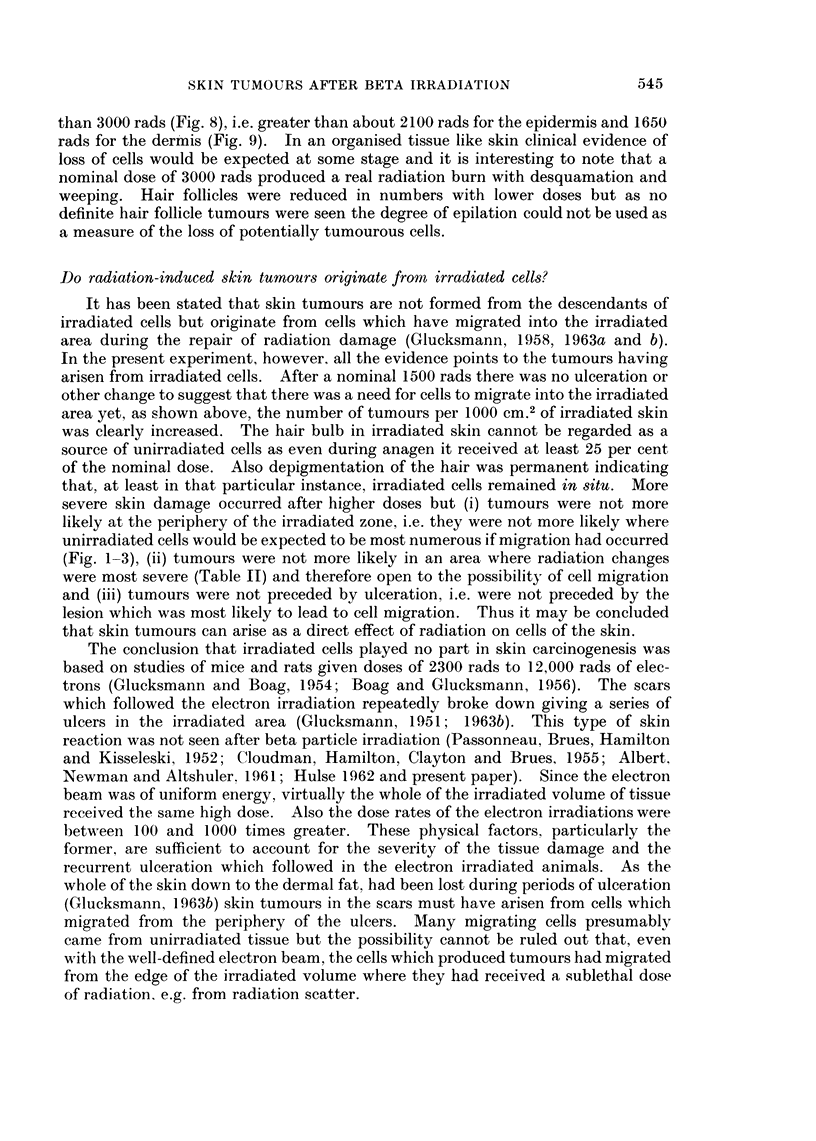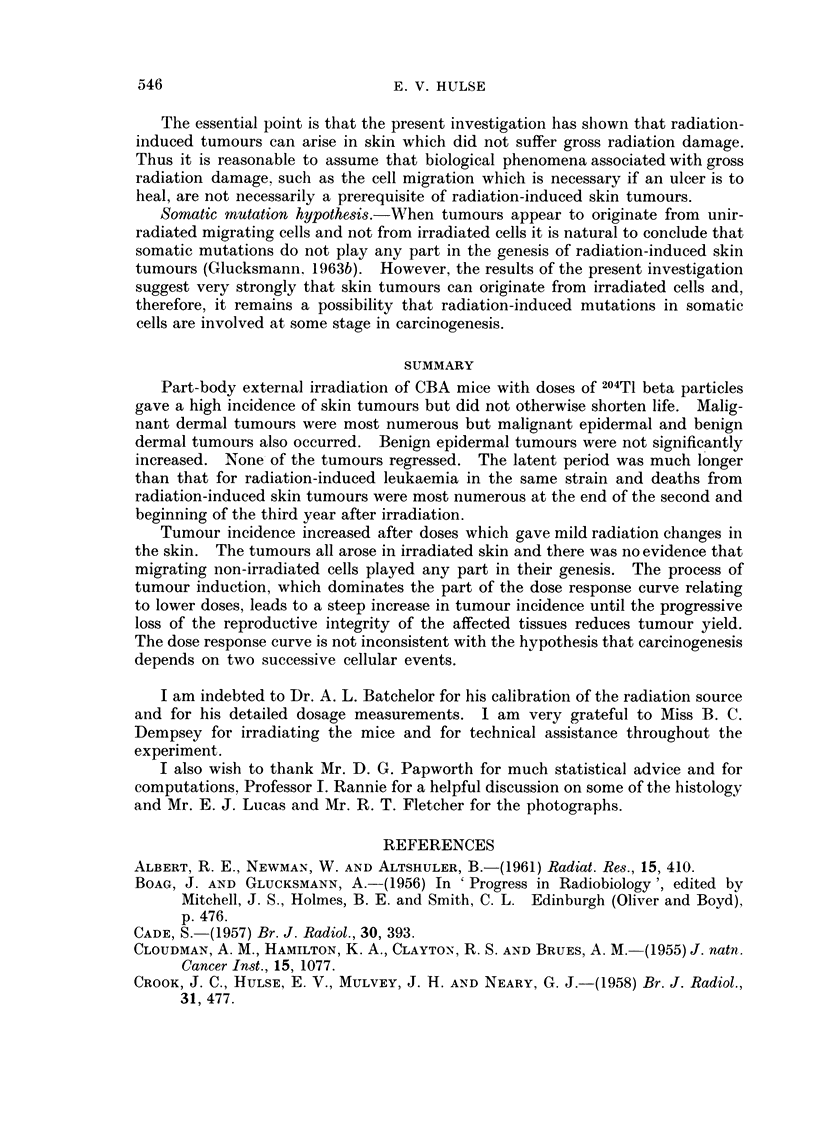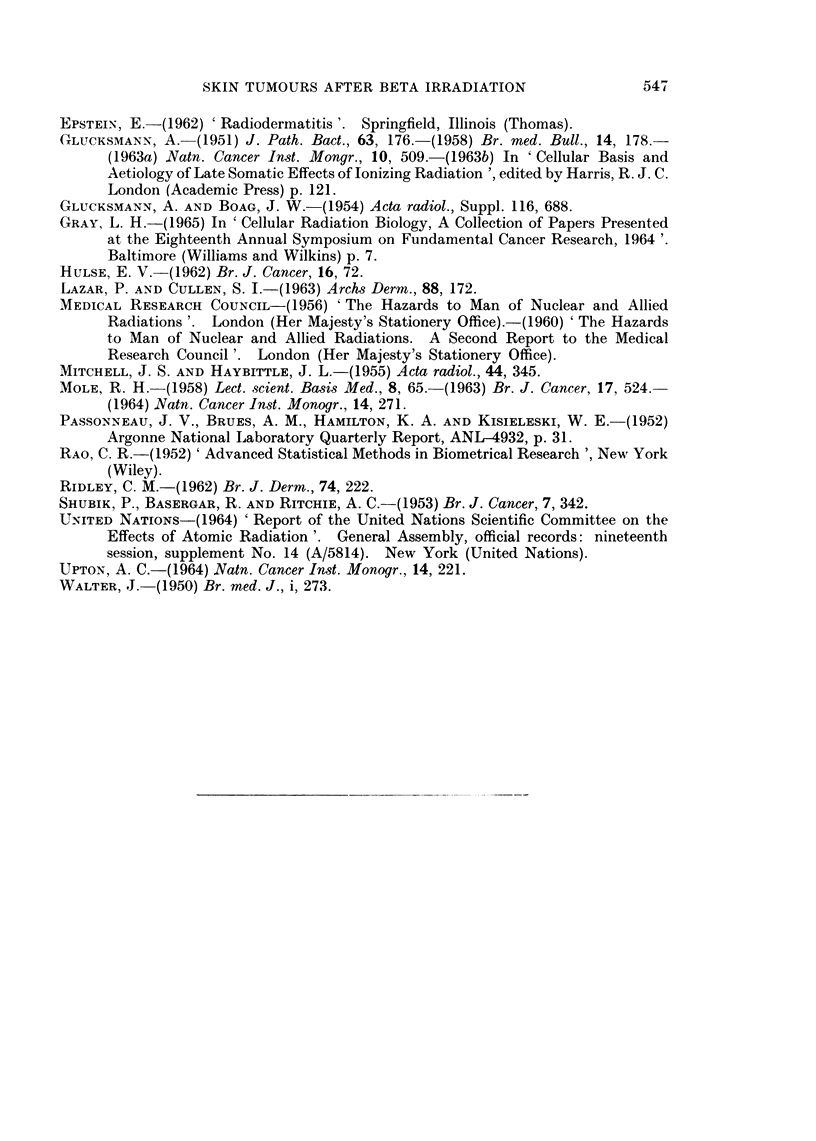# Incidence and pathogenesis of skin tumours in mice irradiated with single external doses of low energy beta particles.

**DOI:** 10.1038/bjc.1967.63

**Published:** 1967-09

**Authors:** E. V. Hulse

## Abstract

**Images:**


### INCIDENCE AND PATHOGENESIS OF SKIN TUMOURS IN MICE IRRADIATED WITH SINGLE EXTERNAL DOSES OF LOW ENERGY BETA PARTICLES

E. V. HULSE

*From the Medical Research Council Radiobiological Research Unit, Harwell, Didcot, Berkshire*

Received for publication December 29, 1966

It is often assumed that severe radiation dermatitis is a prerequisite of radiation-induced skin cancer. Experimentally, a high incidence of skin tumours may follow doses of beta particles large enough to produce a severe radiation burn but there is little information about the carcinogenic effects of lower doses. It was, therefore, decided to extend a previous investigation (Hulse, 1962) to include radiation exposures which produced minimal acute changes and it was found that these also increased the incidence of skin tumours. A dose response curve was obtained which is consistent with the hypothesis that two successive cellular events are needed before a tumour occurs.

In earlier experiments a $^{90}$Sr source was used but the high energy and penetrating power of the beta particles led to complications which killed the mice before tumour incidence was maximal (Hulse, 1962). In the present investigation these were avoided by using the much less penetrating beta particles from $^{204}$Tl.

### METHODS

### *Animals*

420 female CBA/H mice were irradiated and 58 were mock-irradiated. Their ages ranged from two to four months and the mean age at irradiation was three months. The doses of radiation were randomly allocated and no two mice in a litter received exactly the same exposure. They were caged as litters for most of their lives but, to protect the skin during the acute phase of radiation damage, each mouse was housed separately for the period two to six weeks after irradiation. When a tumour developed the mouse was housed separately for the rest of its life. The mice were allowed to die naturally unless they were moribund or the size of tumour or its degree of ulceration made it necessary to kill the animal.

### *Radiation procedure*

The method of Crook, Hulse, Mulvey and Neary (1958) was used. $^{204}$Tl was incorporated into an open-ended cylindrical foil 1·1 cm. long and 2·5 cm. in diameter. The mice were placed in a thin-walled celluloid tube of the same diameter which was then adjusted within the cylindrical foil. The mice fitted snugly within the celluloid tubes and their fur was lightly compressed against the sides. Thus, as the head was never irradiated, the area of skin exposed to the source was the same size as the $^{204}Tl$ cylindrical foil, i.e. 8·6 cm.$^2$ Such an exposed area of skin will be referred to as an irradiated zone. The apparatus was enclosed in a Perspex box and any wriggling movements could be observed and the mouse's position corrected. The mice were supplied with a current of fresh air throughout the irradiation.

*Dose of radiation*

Nominal doses ranged in geometrical progression from 750 rads to 12,000 rads (Table I). Measurements of dose were made at the inner surface of the celluloid tube, i.e. at the surface in contact with the compressed hair of the mouse. The dose-rate decreased from 87 rads/min. to 68 rads/min. during the 21 months when the mice were being irradiated. $^{204}Tl$ beta particles have a relatively low energy (maximum: 0·765 MeV; average 0·24 MeV) and a maximum range in soft tissue of 3 mm. Thus the doses received by the dermis and epidermis were considerably less than the nominal dose. The hair (density 13 mg./cm.$^2$) reduced the dose to 72·5 per cent of the nominal and physical measurements with a small ion chamber and layers of tissue-equivalent material showed that the germinative layer of the epidermis would receive 69–72 per cent and the dermis 40–70 per cent of the nominal doses quoted in Table I. Resting hair bulbs would receive doses within the range quoted for the dermis. However during active growth they may almost reach the panniculus carnosus where the dose would be reduced to 25–39 per cent of the nominal.

*Areas of skin irradiated*

The mice were irradiated either over one zone or two zones in succession. When in the celluloid tube the mouse's body, from neck to root of tail, was slightly longer than the length of three irradiated zones. When a single zone was irradiated the $^{204}Tl$ source was placed about the middle of the trunk. When two zones were irradiated the source was usually positioned over the thorax and over the pelvis with an intervening unirradiated gap of about 1 cm. When the pelvic region was irradiated the hind limbs came within the irradiated zone and the proximal parts of the forelimbs were included in the thoracic zone (as in Fig. 2 of Hulse, 1962). In one group the two zones were arranged to be immediately adjacent, half the group being irradiated over the thorax and mid-trunk and half over the mid-trunk and pelvis. The mice could usually move slightly to and fro within the tube and the positioning could never be absolutely exact so when adjacent zones were irradiated small areas at the junction of the two zones probably received doses somewhat greater than the rest of the zones. Such mice were included to see whether the slight overlapping and, consequently, the increased radiation dose appreciably altered tumour incidence.

There were 58 mock-irradiated mice and further data on the control incidence of skin tumours were obtained from observations on the unirradiated skin of irradiated mice. For subsequent calculations it was presumed that the skin from neck to root of tail in a mock-irradiated mouse was equal in area to three irradiated zones. The unirradiated skin of a mouse irradiated over one zone was presumed to be equal in area to two zones and likewise when two zones were irradiated the unirradiated skin was presumed to be equal in area to one zone. The total numbers of irradiated and unirradiated zones are given in Table I.

TABLE I.—*The Number of Mice Exposed to Various Doses of Beta Particles, the Number of Irradiated Zones, the Number of Unirradiated Zones, the Mean Age at Death of Mice Without Tumours of the Skin or Subcutaneous Tissue and the Percentage of Mice Dying with Skin Tumours*

| Nominal dose in rads | Number of zones irradiated per mouse | Number of mice irradiated | Total number of zones: Irradiated skin | Total number of zones: Unirradiated skin | Mean age at death of mice not having tumours of skin or subcutaneous tissue (months ± S.E.) | Percentage of mice with skin tumours |
|---|---|---|---|---|---|---|
| 12000 | 1 | 30 | 30 | 60 | 25±2 | 57 |
| 6000 | 1 | 31 | 31 | 62 | 26±2 | 42 |
| 3000 | 1 | 60 } | 180 | 180 | { 29±1 | 25 } 38 |
| ,, | 2, separate | 60 } | | | { 24±2 | 50 } |
| 1500 | 2, adjacent | 60 | 120 | 60 | 30±1 | 20 |
| ,, | 1 | 59 } | 179 | 178 | { 27±1 | 7 } 12 |
| ,, | 2, separate | 60 } | | | { 28±1 | 17 } |
| 750 | 2, separate | 60 | 120 | 60 | 29±1 | 3 |
| Mock irradiated | | 58 | 0 | 174 | 29±1 | 0 |
| All dose levels combined | | | 660 | 774 | | |

RESULTS

*Effects on skin*

After a nominal dose of 750 rads the only visible change was a loss of hair pigment. At six weeks after irradiation some depigmented hairs were visible in about half the mice. The depigmentation increased in the ensuing months and at death the hair of the irradiated zone was completely white in nearly all the mice.

After a nominal dose of 1500 rads the hair in the irradiated area became ruffled at the beginning of the third week and began to fall out shortly afterwards. Epilation was never complete and at six weeks patches of partially depigmented hair remained. Regrowth occurred and by the time of death the irradiated zone was covered by white hair in about half the mice. A small patch of permanent epilation was present in the remainder and in two there was a little scaling of the epilated skin. When adjacent zones were given 1500 rads one third of the mice sustained a small radiation burn in the centre of the irradiated area (i.e. where some overlap of the zones was possible). At death the areas of epilation were larger and sometimes completely encircled the animal's body.

Definite radiation burns developed after nominal doses of 3000, 6000 and 12,000 rads. During the second week the hair became ruffled and appeared to " stand on end ". From the beginning of the third week hair was lost revealing a red skin which frequently desquamated leaving a moist surface. During the fourth week the skin dried and in some instances small crusts or scabs formed. Healing was well under way during the fifth and sixth weeks. At six weeks after irradiation the epilated area encircled the body and a small scab or a small scar, indicating at least a second degree burn, was present in about one third of the mice given 3000 rads and four-fifths of the mice given 12,000 rads. Depigmentation was not seen until sometime after the burn had healed. At death the irradiated zone of five of the 120 mice given 3000 rads was sparsely covered with depigmented hair. In all other mice given 3000 rads or more a permanently epilated strip with depigmented hair at the edges encircled the body. Some scaling of the irradiated skin was present in 3–10 per cent of the mice. Only one indolent ulcer occurred and that was seen in a mouse given 3000 rads.

As it is impossible in intact hairy mice to see whether a non-epilating dose produced erythema a small group of albino hairless mice were used to observe the effect of 750 rads and 1500 rads. A nominal dose of 750 rads did not produce any visible change and 1500 rads produced a slight erythema. In previous experiments burns following beta irradiation were more severe in hairless mice than in CBA mice and it was shown that the difference in severity was not due to lack of hair or pigment (Crook, Hulse, Mulvey and Neary, 1958). Thus it may be presumed that in the CBA mice 750 rads was definitely a sub-erythema dose and that if a nominal dose of 1500 rads did produce an erythema it was only very slight.

Skin was taken for histology at autopsy from many of the mice and included skin with the maximum clinical changes for the various doses. Histologically the changes were not uniform throughout the irradiated zone. After nominal doses of 750 or 1500 rads the epidermis was sometimes slightly hyperkeratotic and small patches of either slight thickening or slight thinning of the epidermis were seen. Occasionally after 1500 rads the epidermis was flattened. The dermis was sometimes a little thicker than normal, in which case it frequently tended to be less cellular than usual and the collagen fibres were rather closely packed. However, patches of slightly increased cellularity were sometimes seen.

After nominal doses of 3000 rads and above the changes were more pronounced. Patches of either epidermal thickening or thinning were seen more frequently and in mice with scaling there was hyperkeratosis and parakeratosis. Changes in the dermis ranged from slight thickening to occasional patches of fibrosis, the cellularity of which varied. For the most part the fibrosis was above the level of the panniculus carnosus but sometimes there was a little excess of fibrous tissue below the muscle. Very occasionally venules were dilated but no other changes in vessels were observed. Indeed the irradiation was too superficial for radiation changes in large vessels to be expected.

In order to confirm that the $^{204}$Tl beta particles did not damage deep structures three CBA mice were given 12,000 rads in the region of the sternum and ribs. Multiple examinations of the bone marrow three days later did not reveal any damage to haemopoietic tissue.

*Cause of death*

Each mouse was autopsied and its date of death noted. Previous experiments (Hulse, 1962) suggested that superficial tumours would be the main source of any shortening of life. In the present investigation it was found that all other causes of death were very similar in irradiated and control mice. Mice which received 3000 rads or above tended to die slightly earlier (Table I) but in no instance was the mean age at death of the non-tumour-bearing mice of an irradiated group significantly different from that of the controls. Thus if there was any non-specific life shortening it was very small.

### *Tumours*

### *Types of tumours*

A total of 133 tumours arose in tissues which were or might have been affected by the irradiation and seven tumours arose in similar tissues outside the irradiated zones. Some were ulcerated and others reached a large size without ulcerating. Histologically an ulcerated tumour was not necessarily malignant and conversely a non-ulcertated tumour was not necessarily benign.

*Epidermal tumours.*—Only two of the 20 tumours were benign, both being highly keratinized sessile papillomas. The remaining 18 were squamous cell carcinomas and were all situated on the torso except for one in the region of a knee. Most of the carcinomas were well differentiated.

*Dermal tumours.*—There were 96 tumours of irradiated dermis and 77 of them were malignant. Of these, 74 were fibrosarcomas. Most were well differentiated but 13 of the fibrosarcomas were either anaplastic or very cellular and two contained giant cells. Two fibrosarcomas contained small amounts of bone but it was impossible to tell whether the metaplasia had occurred before or after the tumour appeared in the irradiated skin. In five tumours, parts consisted of well differentiated acellular fibrous tissue suggesting that these particular tumours may have started as fibromas and later became malignant. In four of the malignant tumours of connective tissue the cells were atypical and in some places their appearance suggested a nerve sheath origin. Two were perianal tumours and two arose in irradiated tissue near the ankle. Histologically they resembled the apparently benign, yellowish fusiform tumours very occasionally seen in the tails of unirradiated mice.

Two of the malignant tumours apparently arising in the dermis were haemangioendotheliomas. Another malignant tumour, which presented as a small thickening of the dermis, was found to consist of numerous clefts and cyst-like spaces lined by tumour cells with intervening hyaline connective tissue. Histologically it was reminiscent of a synovioma but it occurred in the dorsal mid-line about the junction of the thorax and lumbar region and did not show any evidence of being related to the synovia of a joint, tendon sheath or bursa.

There were 19 fibromas of the dermis. Most of them were relatively acellular but two were sufficiently cellular to suggest the possibility that they might be in the process of losing their benign character.

*Tumours beneath the dermis.*—Five fibrosarcomas occurred beneath the irradiated skin. One well differentiated and one anaplastic fibrosarcoma arose in subcutaneous connective tissue beneath the panniculus carnosus. One tumour had arisen in close relation to the lumbar vertebrae and the remaining two fibrosarcomas were intra-thoracic. Some 12 breast tumours occurred in irradiated zones and of these one showed squamous metaplasia and one fibrous metaplasia.

*Tumours of unirradiated tissues.*—A total of 7 tumours of the same or related tissues occurred in the unirradiated zones of irradiated mice. One was a breast tumour, four were fibrosarcomas of sub-dermal connective tissue, one was an intrathoracic fibrosarcoma and one was an osteogenic sarcoma of the ribs.

No tumours of the epidermis or dermis were seen in the unirradiated control mice. Breast tumours and subdermal fibrosarcomas were also absent but both are occasionally seen in CBA/H mice, e.g. in the ensuing experiment one breast tumour, one intrathoracic fibrosarcoma and two retroperitoneal fibrosarcomas were found in 60 unirradiated mice.

*Multiple skin tumours.*—Twelve mice developed two or more tumours in irradiated skin and in all but two the nominal dose was 3000 rads. A fibrosarcoma and a squamous cell carcinoma co-existed in five mice. In two of these, quite separate tumours occurred in the same zone and in two others the tumours were in separate zones. In the fifth mouse the superficial part of the growth was a squamous cell carcinoma and the deep part was a fibrosarcoma with no evidence of metaplasia from one type of tissue to the other.

A fibrosarcoma and a haemangioendothelioma occurred in separate zones in one mouse and in another mouse two quite separate fibrosarcomas occurred in separate zones. Both fibrosarcomas and fibromas occurred in four mice, in three the tumours were in separate zones and in one mouse, which had received 12,000 rads, the two quite separate tumours were in the same zone. One mouse, which had received a nominal dose of 1500 rads to two adjacent zones, had three separate small fibromas in the irradiated area.

*Position of tumours in relation to skin damage*

It was always possible to define the area irradiated by the extent of depigmentation. The majority of tumours occurred well within the irradiated area, i.e. following lower doses they were away from the junction of pigmented and depigmented hair and following epilating doses the tumours started in the epilated area and, whilst relatively small, were separated from non-irradiated skin by a fringe of depigmented hair (Fig. 1–3). On a few occasions a tumour appeared to have started at the edge of, but wholly within, the irradiated area, e.g. in the region of the depigmented fringe round an area of epilation.

When a definite scar was visible in the skin at six weeks after irradiation a note was made of its position. The scars were small and situated about the centre of the irradiated zone and any cells which may have migrated into the scarred area during healing must have come from surrounding irradiated skin. None of the scars broke down and with passage of time they became more difficult to see. As the position of each tumour was recorded it was possible to find how many developed in the region of a scar. These findings,for the three doses which produced scarring, are given in Table II from which it can be seen that there is nothing to suggest that the presence of a scar increased the likelihood of a skin tumour.

The one indolent ulcer was not preceded by a scar and was not associated with a tumour. There was no evidence that any tumour was preceded by chronic ulceration though a few tumours did ulcerate when they were quite small. Although the irradiated skin was frequently inspected there was never any evidence of dermal necrosis or other similar change.

---

EXPLANATION OF PLATE

Fig. 1.—A mouse given a nominal dose of 3000 rads to two zones with a small fibrosarcoma in the centre of the epilated area of the right side of the cephalic zone.

Fig. 2.—Same mouse as in Fig. 1 showing a fibroma in the centre of the left side of the caudal zone.

Fig. 3.—Dorsal aspect of a mouse given a nominal dose of 3000 rads to two zones. The small tumour to the left of the mid line in the epilated part of the cephalic zone is a squamous cell carcinoma. Note that the skin is otherwise well healed.

1

2

3

TABLE II.—*Skin Tumours and Their Relation to the Small Areas of Scarring*

| Nominal dose in rads | Number of mice with a scar | Number of tumours at the site of the scar | Number of tumours not at the site of the scar | Number of mice without a scar | Number of tumours in mice without a scar |
|---|---|---|---|---|---|
| 12,000* | 20 | 3 | 12 | 6 | 1 |
| 6000 | 5 | 2 | 0 | 26 | 11 |
| 3000 single zone | 22 | 4 | 2 | 38 | 10 |
| 3000 two zones | 13 | 3 | 6 | 47 | 30 |

* Four mice, two of which developed tumours, are not listed because their skin condition was not recorded immediately after healing of the radiation burn.

*Incidence*

The percentage of mice with tumours (Table I) demonstrates the high incidence obtained. The actual number of tumours developing does not, however, depend only on the number of mice irradiated. A mouse irradiated over two zones is, presumably, more likely to develop a tumour than one irradiated with the same dose over a single zone. A better way of expressing incidence is, therefore, to compare the number of tumours occurring with the total number of zones irradiated. This allows comparisons within the experiment and for the statistical tests referred to below incidence was expressed as tumours per zone.

It might be objected that data from mice irradiated over two zones should be treated separately as a tumour in one zone could shorten life and thus reduce the likelihood of a tumour appearing in the other zone. Alternatively irradiation of two zones might, for some reason, increase the efficiency of the carcinogenic process. In fact the data (Table III) show that this is not so, e.g. there were twice as many malignant dermal tumours in mice given 3000 rads to two zones as in mice given the same dose to a single zone. Even where the numerical difference is greatest, i.e. for malignant epidermal tumours after 3000 rads, the number of tumours after irradiation of two zones was not significantly different from twice the number after single zone irradiations ($P = 0 \cdot 104$ using Rao's (1952) exact test)

TABLE III.—*Number of Skin Tumours after Single Zone Irradiations Compared with Number after Irradiations of Two Separate or Adjacent Zones*

| Nominal dose in rads | Number of zones per mouse | Number of mice | Number of epidermal tumours | | Number of dermal tumours | |
|---|---|---|---|---|---|---|
| | | | Benign | Malignant | Benign | Malignant |
| 3000 | 1 | 60 | 0 | 1 | 3 | 12 |
| ,, | 2 (separate) | 60 | 0 | 9 | 5 | 25 |
| 1500 | 1 | 59 | 1 | 1 | 1 | 1 |
| ,, | 2 (separate) | 60 | 1 | 0 | 2 | 7 |
| ,, | 2 (adjacent) | 60 | 0 | 1 | 6 | 7 |

If the area of one zone is presumed to equal the area of the $^{204}Tl$ source, i.e. $8 \cdot 6$ cm.$^2$, the number of tumours per 1000 cm.$^2$ of irradiated skin can be calculated and comparisons with other experiments are more easily made (Hulse, 1962). Tumour incidence is expressed in this way in Table IV and it is obvious that the incidence of malignant dermal tumours increased with the dose of radiation.

TABLE IV.—*Incidence of Tumours After Beta Irradiation Expressed as Number of Tumours per* 1000 *cm.*² *of Skin. Number of Tumours Actually Observed in Parentheses*

| Nominal dose in rads | Epidermal tumours | | Dermal tumours | | Breast tumours | Subdermal fibrosarcomas |
|---|---|---|---|---|---|---|
| | Benign | Malignant | Benign | Malignant | | |
| 12000 | 0 | 7·7 (2) | 7·7 (2) | 54·1 (14) | 3·9 (1) | 0 |
| 6000 | 0 | 11·2 (3) | 0 | 37·3 (10) | 0 | 0 |
| 3000 | 0 | 6·4 (10) | 5·1 (8) | 23·8 (37) | 1·9 (3) | 0 |
| 1500 adjacent zones | 0 | 0·96 (1) | 5·8 (6) | 6·8 (7) | 1·9 (2) | 1·9 (2) |
| 1500 single or separate zones | 1·3 (2) | 0·65 (1) | 1·9 (3) | 5·2 (8) | 2·6 (4) | 0·65 (1) |
| 750 | 0 | 0·96 (1) | 0 | 0·96 (1) | 1·9 (2) | 1·9 (2) |
| Unirradiated zones in irradiated mice | 0 | 0 | 0 | 0 | 0·19 (1) | 0·96 (5) |
| Unirradiated skin in control mice | 0 | 0 | 0 | 0 | 0 | 0 |
| Total unirradiated skin | 0 | 0 | 0 | 0 | 0·15 (1) | 0·75 (5) |

The increase in incidence of benign dermal tumours and malignant epidermal tumours with radiation dose was not as great but nevertheless was very significant ($P < 10^{-6}$ in both cases). The two benign epidermal tumours both occurred in irradiated mice but the nominal dose, 1500 rads, was relatively low. The possibility had to be considered that the breast tumours and subdermal fibrosarcomas which occurred beneath irradiated skin were actually induced by the radiation but comparisons which control mice and unirradiated zones of irradiated mice did not provide any statistical evidence to suggest that this was so ($P = 0·85$ for breast tumours and 0·44 for subdermal fibrosarcomas).

The incidence of benign dermal and malignant dermal tumours after 1500 rads (single or separated zones) were very significantly different from those in the unirradiated skin ($P = 0·008$ and $P = 2·6 \times 10^{-6}$ respectively, using an exact test similar to that described by Rao (1952)). Dermal tumours after 750 rads were too few for a comparable test.

The number of malignant epithelial tumours after 1500 rads was too small for statistical testing. When the data for malignant epidermal tumours after 750 rads, after 1500 rads and in unirradiated skin were compared, there was no statistical evidence to suggest that the incidence (tumours per zone) was not the same in all three groups ($P = 0·09$).

The increased radiation dose where adjacent zones overlapped during irradiation might have increased the incidence of tumours. The only suggestive numerical evidence concerns benign dermal tumours (Table III) but the difference in their incidence in mice irradiated over separate zones and mice irradiated over two adjacent zones was not statistically significant ($P = 0·3$ using Rao's (1952) exact test).

*Variations in incidence with time after irradiation*

The earliest tumours to appear, a fibrosarcoma after 6000 rads and a squamous cell papilloma after 1500 rads, were each first seen seven months after irradiation at which time mortality in each group ranged from 2–5 per cent, i.e. no more than three mice had died in any one group. Since this loss was so small no correction was made for it and tumour incidences are based on the number of mice irradiated. The first fibroma was seen at 15 months after 12,000 rads and the first squamous cell carcinoma at 19 months after 3000 rads.

The cumulative percentages of mice with skin tumours are given in Fig. 4 and 5.

FIG. 4 (left).—Cumulative percentage of mice with dermal tumours.
FIG. 5 (right).—Cumulative percentage of mice with epidermal tumours.
In both graphs the incidence is plotted at the middle of the six month period to which it refers.

| | |
|---|---|
| ○———○ | Nominal dose of 12,000 rads single zone |
| △———△ | " " " 6000 " " " |
| □———□ | " " " 3000 " " " |
| ▽———▽ | " " " 1500 " " " |
| ■ – – – ■ | " " " 3000 " two separate zones |
| ▼ – – – ▼ | " " " 1500 " " " " |
| ● – – – ● | " " " 750 " " " " |
| ×·–·–·× | " " " 1500 " " adjacent " |

The age-specific death rates for mice with skin tumours are shown in Fig. 6 and 7 where tumour incidences amongst decedents are given as the number of tumours per 1000 cm.$^2$ of skin irradiated and not as the number of mice with tumours. As there was histological evidence that fibromas might become fibrosarcomas the data for benign and malignant dermal tumours have been combined.

The maximum incidence of dermal tumours in all dose groups occurred during the third year after irradiation. The first mice to die with dermal tumours had received a nominal dose of at least 3000 rads and there is a slight tendency for the curves for lower dose groups to be shifted to the right (Fig. 6). The 12,000 rad and 6000 rad mice which survived into the second half of the third year after irradiation did not have dermal tumours but the 3000 rad and 1500 rad mice did and this again suggests that as the dose is decreased the tumours occur later in time.

Epidermal tumours were comparatively few and only the three highest doses are illustrated in Fig. 7. Mice did not start dying with malignant epidermal

FIG. 6 (left).—Age-specific mortality from dermal tumours. The number of tumours per 1000 cm² was calculated for six month intervals and is plotted at the middle of the six month period to which it refers.

FIG. 7.—Age-specific mortality from epidermal tumours. Presentation as in Fig. 4. There were few tumours after 750 rads and 1500 rads and for clarity these have been omitted. The incidence as 27 months after 12,000 rads (indicated by an arrow) was 57·8 tumours per 1000 cm² (see text).

| | | | |
|---|---|---|---|
| O———O | Nominal dose of 12,000 rads, single zones | | |
| Δ———Δ | " " " | 6000 " | " " |
| □— — —□ | " " " | 3000 " | single or two separate zones |
| X·—·—·—X | " " " | 1500 " | two adjacent zones |
| ∇— — —∇ | " " " | 1500 " | single or two separate zones |
| ●— — —● | " " " | 750 " | two separate zones |

tumours until the latter half of the second year after irradiation, i.e. from six to eighteen months after those with malignant dermal tumours. Their peak incidence is about the same time as that of the dermal tumours but is less clearly defined. The high terminal incidence after 12,000 rads is due to one of the two surviving mice dying with a tumour.

*Dose response relationships*

The data were examined to see whether they are compatible with a linear arithmetic increase in incidence with dose, presuming a non-threshold response with zero incidence in the controls. Such a relationship was not incompatible with the data for malignant epidermal tumours ($\chi^2$ = 3·54; P = 0·17; b = 2·35 ($\pm$0·57) $\times$ $10^{-5}$) and benign dermal tumours ($\chi^2$ = 2·78; P = 0·094; b = 1·80 ($\pm$0·50) $\times$ $10^{-5}$) but the data for malignant dermal tumours departed significantly from a linear relationship ($\chi^2$ = 9·79; P = 0·044).

Inspection of the data for malignant dermal tumours suggested that their incidence could be related to the logarithm of the dose. By using the method of successive approximations assuming a Poisson distribution of tumours per zone

FIG. 8.—Incidence of dermal and epidermal tumours after beta radiation (single or separate zones). The incidence is given as number of tumours per zone of skin irradiated and the standard errors were calculated assuming a Poisson distribution of tumours per zone. The doses are nominal surface doses, not the doses within the epidermis or dermis which are considerably less due to the low penetrating qualities of the radiation. The initial portions of both curves and their extrapolations are plotted assuming that incidence is proportional to the square of the dose.

○——○ Dermal tumours.

●·—·—·● Epidermal tumours.

it was found that a straight line provided a satisfactory fit (P = 0·09; a = 0·79 $\pm$ 0·11; b = 0·31 $\pm$ p·04). The data for malignant epidermal tumours, being the next most numerous, were then examined in the same way and it was found that a straight line again provided a satisfactory fit (P = 0·27; a = $-$0·17 $\pm$ 0·06; b = 0·069 $\pm$ 0·023).

Further possibilities are curvilinear relationships such as those illustrated in Fig. 8. The incidence of dermal tumours increases markedly over the range 750–3000 rads but the increase is less marked after higher doses. The variation in incidence of epidermal tumours follows a similar pattern.

The efficiency with which beta irradiation produces tumours decreases as the dose increases (Fig. 8). This effect is more obvious in Fig. 9 where tumour yield, calculated as number of tumours per 1000 cm.[2] per 1000 rads, is plotted against dose. In this figure allowances have been made for alterations in the dose due to the depth of the target tissue and the actual doses to the germinal layer of the epidermis and to the dermis have been presumed to be about the middle of the ranges quoted earlier, in the section on methods. From Fig. 9 it can be seen that

Fig. 9.—Yield of dermal and epidermal tumours per rad after various doses of beta particles (single or separate zones). The doses were calculated presuming that the amount of radiation received by the germinative zone of the epidermis was 70 per cent and the average dose to the dermis 55 per cent of the nominal doses (see text).

O———O Dermal tumours.

●·—·—·—·● Epidermal tumours.

the yield per rad of dermal tumours increases rapidly and linearly to 1650 rads, i.e. to a nominal dose of 3000 rads, but falls off considerably with higher doses. Epidermal tumours show a similar pattern with a peak yield at 2100 rads, which again is equivalent to a nominal dose of 3000 rads.

### DISCUSSION

### *Types of tumour*

Radiation increased the incidence of benign and malignant dermal tumours and of malignant epidermal tumours but not of benign epidermal tumours (Tables I and IV). Malignant dermal tumours were much the most numerous and most of these were fibrosarcomas. Histologically some seemed to have started as fibromas but the majority appeared to arise as sarcomas *de novo*. The malignant epidermal tumours, which were all squamous cell carcinomas, were either warty or ulcerated growths and there was no histological evidence to suggest an origin in hair follicles or other adnexa.

There was also a preponderance of malignant dermal tumours in the previous investigation (Hulse, 1962). As noted at that time some investigators have found more sarcomas than carcinomas, some more carcinomas than sarcomas and some equal numbers of each type. The variations do not seem to be due to species differences or to differences in the type of radiation.

It is interesting to note that papillomas of the skin which are common in mice after the repeated applications of chemical carcinogens (Shubik, Baserga and Ritchie, 1953) were very rare after beta irradiation. Also tumours which appear after a single dose of radiation do not regress whereas they do after a single application of a chemical carcinogen.

*Time interval between irradiation and tumour formation*

Dermal tumours appeared sooner than epidermal tumours but in both cases high incidences did not occur until the end of the second and beginning of the third year after irradiation (Fig. 6 and 7). Tumours tended to appear earlier after the higher doses, as occurred previously (Hulse, 1962). Although human skin cancer can occur soon after irradiation (Walter, 1950) the latent period in man usually lasts many years, even as long as half a century (Mitchell and Haybittle, 1955; Cade, 1957; Ridley, 1962). This contrasts markedly with radiation induced leukaemia the latent period of which in man is much less and is commonly under ten years (Medical Research Council, 1960; United Nations, 1964). In this respect it is of interest to note that maximum mortality from leukaemia in mice of the same strain and sex given 1000–2000 r of $^{60}$Co gamma-rays was nine to twelve months after irradiation (Mole, 1964). Thus in the mouse and in man the latent period for radiation induced skin tumours is much longer than that for radiation induced leukaemia.

*Incidence*

In the present experiment the total tumour incidence was 69·5, 48·5 and 35·3 per 1000 cm.$^2$ for nominal doses of 12,000 rads, 6000 rads and 3000 rads respectively. These values are higher than the 21 per 1000 cm.$^2$ for nominal doses of 3,300–24,000 rads obtained in earlier experiments using the same strain of mice (Hulse, 1962) but in those experiments many mice died early in the induction period because the small intestine has been seriously damaged by the highly penetrating beta particles from the $^{90}$Sr source which was used at that time.

Experiments cannot demonstrate whether or not there is a threshold dose of radiation below which no tumours are induced (Mole, 1958). However, a significant increase in dermal tumours followed a nominal dose of 1500 rads which would give an actual dose of 600 rads to the deepest layer of the dermis and 1050 rads to its most superficial layer. Thus if a threshold exists for skin tumours in the mouse it must be below 1050 rads. The data can be made to fit a non-threshold type of response, as seen in Fig. 8, and in the equation for the arithmetic dose response curves, quoted above, but the number of tumours after a nominal 750 rads is too small for valid statistical comparisons with the control data.

The vast majority of radiation-induced skin tumours in man are associated with a degree of skin damage which suggests a dose of radiation of several thousand roentgens (Medical Research Council, 1956). There are, however, a few reports of tumours appearing in skin which had been irradiated but which clinically appeared normal (Ridley, 1962; Lazar and Cullen, 1963). The present investigation shows that a definite statistically significant increase in incidence can follow a dose of radiation which, in the acute phase, gives no more than slight erythema and partial epilation and which, in the latter stages, gives a little epilation and only very rarely indeed gives any chronic scaling. Thus, in the mouse at least, cancer of the skin can follow a dose of radiation which does not produce the sort of skin condition which would normally be classed as chronic radiation dermatitis (Epstein, 1962).

*Tumour induction and tumour yield*

A previous review of published data showed that the incidence of skin tumours after beta irradiation did not alter a great deal over doses ranging from 3000–24,000 rads (Hulse, 1962). This is consistent with the levelling off of the dose response curves with high doses seen in the present experiments (Fig. 8).

Fig. 8 and 9 show that the higher doses of radiation were less effective at producing skin tumours. Such a falling off in efficiency is not uncommon in radiation carcinogenesis and was examined recently by Gray (1965). He suggested that curves such as those illustrated in Fig. 8 are the outcome of two processes, each the result of irradiation. They are (i) the transformation of cells into cancer cells, i.e. " induction " and (ii) a reduction in the number of cells available to produce tumours, called by Gray " loss of reproductive integrity ". The induction process predominates after lower doses but after higher doses there is a decrease in the number of cells available to produce tumours. Thus the efficiency of tumour production would be expected to fall once the dose exceeded some optimum value. In Fig. 8 the lower parts of the curves, which relate to the induction process, have been extrapolated to demonstrate both the type of relationship involved and the falling off in efficiency with higher doses.

*Induction process.*—Gray suggested that tumour induction obeys a power law, i.e. tumour incidence is proportional to the power of the dose. In Fig. 8 the parts of the curves relating to induction and their extrapolations, have been plotted assuming that incidence is proportional to the square of the dose and it can be seen that this is a reasonable description of the data. This relationship is again illustrated in Fig. 9 where tumour yield, i.e. incidence divided by dose, increases linearly, indicating a square law.

Experimental data on the induction of leukaemia by radiation suggest that more than one cellular event is required (Upton, 1964) and evidence from a variety of radiation-induced tumours has led to the hypothesis that two successive cellular events are necessary (Mole, 1963; 1964). The square law responses observed in the present experiment are consistent with the two-event hypothesis. The present data are not suitable for assessing whether the first or second event may be more easily caused by the radiation.

*Loss of reproductive integrity*

The decline in tumour yield after higher doses (Fig. 8 and 9) demonstrates " loss of reproductive integrity " (Gray, 1965). The radiation decreases the number of cells available to produce tumours and thus incidence and yield both fall. The yield of dermal and epidermal tumours decreased for nominal doses greater than 3000 rads (Fig. 8), i.e. greater than about 2100 rads for the epidermis and 1650 rads for the dermis (Fig. 9). In an organised tissue like skin clinical evidence of loss of cells would be expected at some stage and it is interesting to note that a nominal dose of 3000 rads produced a real radiation burn with desquamation and weeping. Hair follicles were reduced in numbers with lower doses but as no definite hair follicle tumours were seen the degree of epilation could not be used as a measure of the loss of potentially tumourous cells.

*Do radiation-induced skin tumours originate from irradiated cells?*

It has been stated that skin tumours are not formed from the descendants of irradiated cells but originate from cells which have migrated into the irradiated area during the repair of radiation damage (Glucksmann, 1958, 1963*a* and *b*). In the present experiment, however, all the evidence points to the tumours having arisen from irradiated cells. After a nominal 1500 rads there was no ulceration or other change to suggest that there was a need for cells to migrate into the irradiated area yet, as shown above, the number of tumours per 1000 cm.$^2$ of irradiated skin was clearly increased. The hair bulb in irradiated skin cannot be regarded as a source of unirradiated cells as even during anagen it received at least 25 per cent of the nominal dose. Also depigmentation of the hair was permanent indicating that, at least in that particular instance, irradiated cells remained *in situ*. More severe skin damage occurred after higher doses but (i) tumours were not more likely at the periphery of the irradiated zone, i.e. they were not more likely where unirradiated cells would be expected to be most numerous if migration had occurred (Fig. 1–3), (ii) tumours were not more likely in an area where radiation changes were most severe (Table II) and therefore open to the possibility of cell migration and (iii) tumours were not preceded by ulceration, i.e. were not preceded by the lesion which was most likely to lead to cell migration. Thus it may be concluded that skin tumours can arise as a direct effect of radiation on cells of the skin.

The conclusion that irradiated cells played no part in skin carcinogenesis was based on studies of mice and rats given doses of 2300 rads to 12,000 rads of electrons (Glucksmann and Boag, 1954; Boag and Glucksmann, 1956). The scars which followed the electron irradiation repeatedly broke down giving a series of ulcers in the irradiated area (Glucksmann, 1951; 1963*b*). This type of skin reaction was not seen after beta particle irradiation (Passonneau, Brues, Hamilton and Kisseleski, 1952; Cloudman, Hamilton, Clayton and Brues, 1955; Albert, Newman and Altshuler, 1961; Hulse 1962 and present paper). Since the electron beam was of uniform energy, virtually the whole of the irradiated volume of tissue received the same high dose. Also the dose rates of the electron irradiations were between 100 and 1000 times greater. These physical factors, particularly the former, are sufficient to account for the severity of the tissue damage and the recurrent ulceration which followed in the electron irradiated animals. As the whole of the skin down to the dermal fat, had been lost during periods of ulceration (Glucksmann, 1963*b*) skin tumours in the scars must have arisen from cells which migrated from the periphery of the ulcers. Many migrating cells presumably came from unirradiated tissue but the possibility cannot be ruled out that, even with the well-defined electron beam, the cells which produced tumours had migrated from the edge of the irradiated volume where they had received a sublethal dose of radiation, e.g. from radiation scatter.

The essential point is that the present investigation has shown that radiation-induced tumours can arise in skin which did not suffer gross radiation damage. Thus it is reasonable to assume that biological phenomena associated with gross radiation damage, such as the cell migration which is necessary if an ulcer is to heal, are not necessarily a prerequisite of radiation-induced skin tumours.

*Somatic mutation hypothesis.*—When tumours appear to originate from unirradiated migrating cells and not from irradiated cells it is natural to conclude that somatic mutations do not play any part in the genesis of radiation-induced skin tumours (Glucksmann, 1963*b*). However, the results of the present investigation suggest very strongly that skin tumours can originate from irradiated cells and, therefore, it remains a possibility that radiation-induced mutations in somatic cells are involved at some stage in carcinogenesis.

### SUMMARY

Part-body external irradiation of CBA mice with doses of $^{204}$Tl beta particles gave a high incidence of skin tumours but did not otherwise shorten life. Malignant dermal tumours were most numerous but malignant epidermal and benign dermal tumours also occurred. Benign epidermal tumours were not significantly increased. None of the tumours regressed. The latent period was much longer than that for radiation-induced leukaemia in the same strain and deaths from radiation-induced skin tumours were most numerous at the end of the second and beginning of the third year after irradiation.

Tumour incidence increased after doses which gave mild radiation changes in the skin. The tumours all arose in irradiated skin and there was no evidence that migrating non-irradiated cells played any part in their genesis. The process of tumour induction, which dominates the part of the dose response curve relating to lower doses, leads to a steep increase in tumour incidence until the progressive loss of the reproductive integrity of the affected tissues reduces tumour yield. The dose response curve is not inconsistent with the hypothesis that carcinogenesis depends on two successive cellular events.

I am indebted to Dr. A. L. Batchelor for his calibration of the radiation source and for his detailed dosage measurements. I am very grateful to Miss B. C. Dempsey for irradiating the mice and for technical assistance throughout the experiment.

I also wish to thank Mr. D. G. Papworth for much statistical advice and for computations, Professor I. Rannie for a helpful discussion on some of the histology and Mr. E. J. Lucas and Mr. R. T. Fletcher for the photographs.

### REFERENCES

ALBERT, R. E., NEWMAN, W. AND ALTSHULER, B.—(1961) *Radiat. Res.*, **15**, 410.

BOAG, J. AND GLUCKSMANN, A.—(1956) In ‘ Progress in Radiobiology ’, edited by Mitchell, J. S., Holmes, B. E. and Smith, C. L. Edinburgh (Oliver and Boyd), p. 476.

CADE, S.—(1957) *Br. J. Radiol.*, **30**, 393.

CLOUDMAN, A. M., HAMILTON, K. A., CLAYTON, R. S. AND BRUES, A. M.—(1955) *J. natn. Cancer Inst.*, **15**, 1077.

CROOK, J. C., HULSE, E. V., MULVEY, J. H. AND NEARY, G. J.—(1958) *Br. J. Radiol.*, **31**, 477.

EPSTEIN, E.—(1962) ‘ Radiodermatitis ’. Springfield, Illinois (Thomas).
GLUCKSMANN, A.—(1951) *J. Path. Bact.*, **63**, 176.—(1958) *Br. med. Bull.*, **14**, 178.—(1963*a*) *Natn. Cancer Inst. Mongr.*, **10**, 509.—(1963*b*) In ‘ Cellular Basis and Aetiology of Late Somatic Effects of Ionizing Radiation ’, edited by Harris, R. J. C. London (Academic Press) p. 121.
GLUCKSMANN, A. AND BOAG, J. W.—(1954) *Acta radiol.*, Suppl. 116, 688.
GRAY, L. H.—(1965) In ‘ Cellular Radiation Biology, A Collection of Papers Presented at the Eighteenth Annual Symposium on Fundamental Cancer Research, 1964 ’. Baltimore (Williams and Wilkins) p. 7.
HULSE, E. V.—(1962) *Br. J. Cancer*, **16**, 72.
LAZAR, P. AND CULLEN, S. I.—(1963) *Archs Derm.*, **88**, 172.
MEDICAL RESEARCH COUNCIL—(1956) ‘ The Hazards to Man of Nuclear and Allied Radiations ’. London (Her Majesty’s Stationery Office).—(1960) ‘ The Hazards to Man of Nuclear and Allied Radiations. A Second Report to the Medical Research Council ’. London (Her Majesty’s Stationery Office).
MITCHELL, J. S. AND HAYBITTLE, J. L.—(1955) *Acta radiol.*, **44**, 345.
MOLE, R. H.—(1958) *Lect. scient. Basis Med.*, **8**, 65.—(1963) *Br. J. Cancer*, **17**, 524.—(1964) *Natn. Cancer Inst. Monogr.*, **14**, 271.
PASSONNEAU, J. V., BRUES, A. M., HAMILTON, K. A. AND KISIELESKI, W. E.—(1952) Argonne National Laboratory Quarterly Report, ANL-4932, p. 31.
RAO, C. R.—(1952) ‘ Advanced Statistical Methods in Biometrical Research ’, New York (Wiley).
RIDLEY, C. M.—(1962) *Br. J. Derm.*, **74**, 222.
SHUBIK, P., BASERGAR, R. AND RITCHIE, A. C.—(1953) *Br. J. Cancer*, **7**, 342.
UNITED NATIONS—(1964) ‘ Report of the United Nations Scientific Committee on the Effects of Atomic Radiation ’. General Assembly, official records: nineteenth session, supplement No. 14 (A/5814). New York (United Nations).
UPTON, A. C.—(1964) *Natn. Cancer Inst. Monogr.*, **14**, 221.
WALTER, J.—(1950) *Br. med. J.*, i, 273.